# Emerging role of exosomes in the pathology of chronic obstructive pulmonary diseases; destructive and therapeutic properties

**DOI:** 10.1186/s13287-022-02820-4

**Published:** 2022-04-04

**Authors:** Hadi Rajabi, Nur Konyalilar, Sinem Erkan, Deniz Mortazavi, Seval Kubra Korkunc, Ozgecan Kayalar, Hasan Bayram, Reza Rahbarghazi

**Affiliations:** 1 Koç University Research Center for Translational Medicine (KUTTAM), Koç University School of Medicine, Istanbul, Turkey; 2 Department of Pulmonary Medicine, School of Medicine, Koç University, Istanbul, Turkey; 3 Stem Cell Research Center, Tabriz University of Medical Sciences, Tabriz, Iran; 4 Department of Applied Cell Sciences, Faculty of Advanced Medical Sciences, Tabriz University of Medical Sciences, Tabriz, Iran

**Keywords:** COPD, Immune cells, Exosomes, Pathological remodeling, Tissue regeneration

## Abstract

Chronic obstructive pulmonary disease (COPD) is known as the third leading cause of human death globally. Enhanced chronic inflammation and pathological remodeling are the main consequences of COPD, leading to decreased life span. Histological and molecular investigations revealed that prominent immune cell infiltration and release of several cytokines contribute to progressive chronic remodeling. Recent investigations have revealed that exosomes belonging to extracellular vesicles are involved in the pathogenesis of COPD. It has been elucidated that exosomes secreted from immune cells are eligible to carry numerous pro-inflammatory factors exacerbating the pathological conditions. Here, in this review article, we have summarized various and reliable information about the negative role of immune cell-derived exosomes in the remodeling of pulmonary tissue and airways destruction in COPD patients.

## Introduction

COPD is a chronic inflammatory condition with progressive bronchopneumonitis, leading to difficulty breathing and limitation of daily tasks [1]. Recent works established the importance and critical role of innate and adaptive immune systems in the pathology of COPD [2]. Prolonged inflammatory response accounts for excessive mucus production, generation of emphysematous foci, obstruction/narrowing of airways, and remodeling of the extracellular matrix (ECM) within the lung parenchyma [3]. The innate immune system response is stimulated in COPD patients coincides with the expression of cytokines such as interleukin-8 (IL-8), matrix metalloproteinase protein-9 (MMP-9), and neutrophil elastase (NE) in certain micro-anatomical regions of pulmonary parenchyma and intra-alveolar septum. These features may associate with reduced airflow capacity and gas exchange between blood and respiratory system [4]. Regardless of the presence of different subsets of immune cells in the COPD pulmonary niche, it is believed that the release of degrading enzymes and inflammatory mediators can regulate the bioactivity of other cells in close or remote sites [5]. Previous works have provided evidence of exosomal cytokines and modulatory effects under pathological conditions [6]. For example, several cytokines have been indicated inside the lumen of Exo released from inflammatory cells, progenitors, and certain stem cell types. Unlike several types of immune cells, mesenchymal stem cells (MSCs) can produce extracellular vesicles (EVs) with a large content of anti-inflammatory cytokines compared to the other cell lineages [7].

Exo are known as nano-sized communication vehicles, playing an interesting role in the paracrine activity of almost all cells under physiological and pathological conditions [8, 9]. Exo can cross all-natural barriers within the body and transfer their cargo to remote sites [6]. Their communicative facilities and components such as microRNAs (miRNAs) make key players in the dynamic activity of cells under pathological conditions [10, 11]. Besides, the critical role of Exo inside the body, using them directly or as a delivery agent is an undeniable part of almost the majority of experimental and clinical studies [12–14]. As expected, immune cells like other cells can actively secret Exo for reciprocal communication [15]. Exo exchange between the immune cells can be assessed from two distinct aspects. Whether and how these Exo can exacerbate the inflammation and remodeling process or trained immune cell Exo may contain certain growth factors that accelerate the regeneration of injured pulmonary microenvironment is the subject of area. It seems that the cellular source and cargo type is determinant in Exo activity under pathological conditions. For instance, under pathological conditions such as sepsis macrophages can release Exo with the ability to increase the expression of intercellular adhesion molecule-1 (ICAM-1) in alveolar epithelial cells and trafficking of immune cells from the blood side into the pulmonary niche, leading to subsequent tissue damage and deleterious outcomes [16, 17]. Unlike these effects, inflamed monocyte-derived Exo contain mitochondrial-associated DAMPs which can diminish neutrophil infiltration into injured sire via the suppression of Toll-like receptor-9 (TLR-9) [18]. It can be hypothesized immune cell Exo possess pleiotropic properties related to pathological state and intensity of inflammation [19]. In this review article, we collected comprehensive information about the role of immune- and stem cell-derived Exo on the pathology of COPD disease.

## Exosome biogenesis

EVs are distributed in different biofluids and are involved in paracrine cross talk between the cells in higher organisms [20]. Generally, the term EVs include a heterogeneous population of vesicles shed by the majority of cell types and can be detected in biofluids. Based on ultrastructural analyses, EVs are classified into different subsets based on size, mechanism of biogenesis, density, function, and origins [21]. EVs include Exo, microvesicles, and apoptotic bodies [22]. Unlike Exo, microvesicles, and apoptotic bodies are directly generated via the protrusion plasma membrane, and their sizes are ranged between 500 to 2000 nm [23]. Among all subtypes of EVs, Exo, with a mean diameter of 40 to 160 nm, are classified as the smallest vesicles with an endosomal origin [24]. In physiological and pathological conditions, several factors such as proteins, nucleic acids (including mRNA and miRNA), viral genetic materials, and lipids are sorted into the Exo lumen, harbored in biofluids, and transferred to the nearby acceptor cells or distant sites [25].

**Fig. 1:**
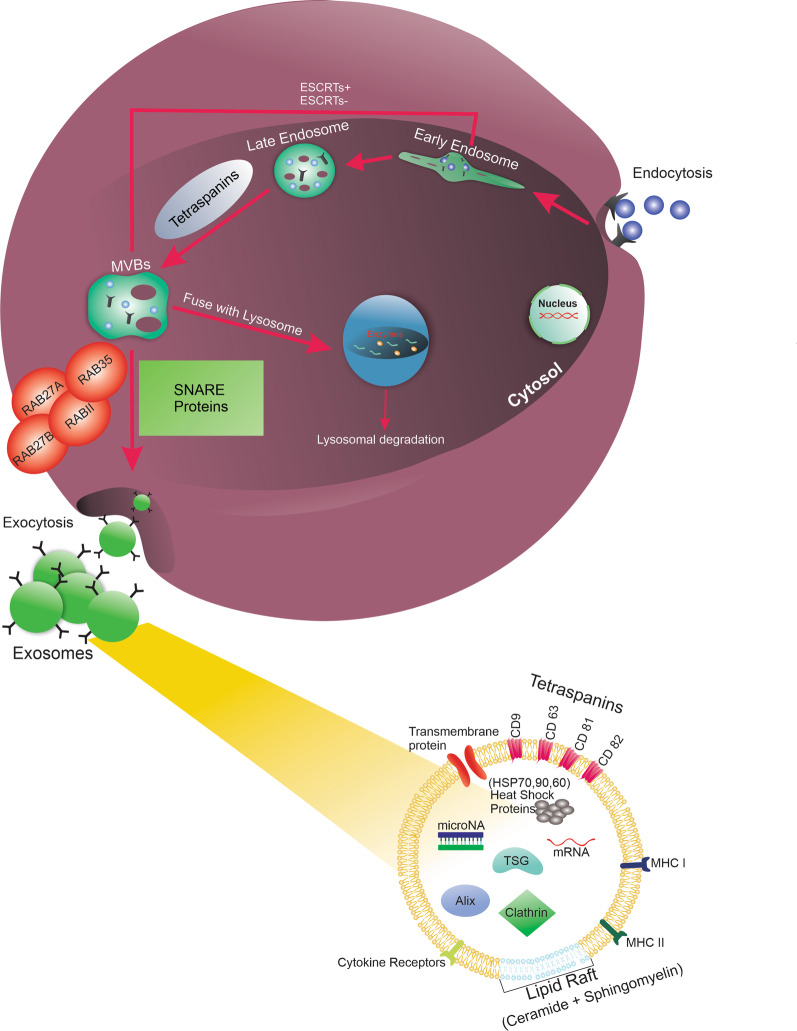
Exo biogenesis and abscission mechanisms. Early endosomes are generated through the invagination of cell membranes. Then, by the inward budding of the vesicle, late endosomes and MVBs are formed. 2 pathways are involved in the exosome biogenesis: ESCRT-dependent and ECRT-independent pathways. Tetraspanins are thought to have a fundamental role in the ECRT-independent pathway. At the end of the exosome biogenesis process, formed MVBs either degraded into lysosomes or fuse with the plasma membrane. As a result of this fusion process, they are released by exocytosis through SNARE proteins and RAB GTPases. Released vesicles are called exosomes. MVB: multi-vesicular body, ESCRT: endosomal sorting complex transport, Rab: Ras-associated binding proteins, TSG: tumor necrosis factor (TNF)-stimulated gene, MHC: major histocompatibility complex

Exo appear relatively spherical and are enclosed by the lipid bilayer membrane, making them stable bioshuttles [26]. One reason would be that distinct factors such as tetraspanins (CD9, 63, 81, and 82), microvesicular bodies (MVB) biogenesis-associated proteins [ALG-2 interacting protein X (Alix)], tumor susceptibility gene 101 (TSG101), clathrin), tumor necrosis factor receptor-1 (TNFR-1), flotillin, docking, and membrane fusion proteins [RABs, adenosine diphosphate ribosylation factor (ARF)], and heat shock proteins [HSPs (Hsp90, Hsp70, and Hsp60)] are tightly attached to Exo membrane during biogenesis [27]. Besides proteins, several lipid elements such as sphingomyelin, cholesterol, ganglioside GM3, and ceramides are distributed in the Exo membrane. The amount of Exo membrane lipids and protein can be different in terms of cell origin and physiological and pathological conditions [28]. In collaboration with ESCRT machinery, lipids can participate in cargo sorting, Exo secretion, and induction of specified signaling pathways in acceptor cells [26, 29]. Nucleic acids such as mRNA, microRNA (miRNA), non-coding RNA, and DNA are other important elements sorted into Exo lumen [26, 29]. In the cytosol, Exo are formed inside MVBs and an endosomal compartment with the collaboration of several machinery systems (Fig. 1) [26, 29]. In a very simple language, the Exo biogenesis consists of MVB formation, intraluminal budding, and cargo sorting. The invagination of cell membrane leads to the generation of early endosomes and further morphological changes lead to inward budding at the vesicle membrane and the formation of late endosomes and MVBs, respectively [30]. Numerous intraluminal vesicles (ILVs), Exo ancestors, are seen inside the MVBs. In the next step, MVBs can fuse with lysosomes or follow the endocytic/exocytic pathway where the membrane of MVBs coalesce with the plasma membrane and protrude the ILVs into the ECM hereafter referred to as Exo [31]. It was suggested that Exo biogenesis happens via two distinct pathways including endosomal sorting complex transport (ESCRT) required for transport (ESCRT)-dependent and -independent mechanisms [32]. The ESCRT system is composed of four different proteins ESCRT-0, -1, -2, and -3 [33]. These proteins are in close contact with other factors like vascular protein sorting associated protein-4 (VPS4), vesicle trafficking 1 protein (VTA1), and ALIX to promote MVBs. Of note, ESCRT-0 and -1 belonging to the ESCRT system possess ubiquitin domains with the ability to recognize ubiquitinated protein and sort into the ILVs lumen [32]. Following cargo sorting, ESCRT-2 and -3 are recalled accelerating intraluminal budding via enzymatic de-ubiquitination of cytosolic proteins which leads to MVBs formation. The activation of the latter protein (ESCRT-3) is also associated with the recycling of the ESCRT system [33]. Suppression of the ESCRT complex does not completely inhibit the Exo biogenesis which is the main reason for the existence of the ESCRT-independent system. Interestingly, in the absence of ESCRT-0, -1, -2, and -3, the process of intraluminal budding continues, indicating an alternative pathway involved in Exo biogenesis [33]. In the ESCRT-independent pathway, the vesicle formation is orchestrated by the regulation of lipid and cargo domains via engaging HSPs, tetraspanins and lipids [34]. Along with these mechanisms, a recently introduced pathway so-called lipid raft participates in Exo biogenesis inside the cells [35]. In this pathway, changes are initiated in the lipid composition of the endosomal compartment, leading to lipid clustering, known as lipid rafts, allowing vesicle formation and intraluminal budding. It is thought that both flotillins and tetraspanins are involve in this mechanism [35]. The Exo secretion in this pathway is not affected by the downregulation of ESCRT components such as hepatocyte growth factor-regulated tyrosine kinase substrate (Hrs), Alix, or Tsg101 proteins, proving that this pathway is not associated with the ESCRT-dependent pathway [35].

Once MVBs are formed, they harbor Exo in their membranes. To be specific, Exo have two distinct directions. They could either be degraded by the lysosomes or fused with plasma membrane resulting in the release of Exo into the ECM. If the MVB is targeted for lysosomal degradation, it will fuse with the lysosome and result in the release of the internal Exo and the macromolecules contained within them, into the lumen of the lysosome. These components will later be exposed to the hydrolytic enzymes and be degraded [36]. As a second fate, the MVBs will move to the plasma membrane and release their ILVs to the extracellular environment. There is molecular machinery involved in the transportation of MVBs to the cell periphery and fusion with the plasma membrane. This molecular machinery mediates the secretion of Exo [30].

Some studies proved that pathways for the secretion of Exo are mediated by Rab GTPases [37, 38]. Even though the mechanism has not been fully understood yet, it has been shown that a GTPase, RAL-1, mediates the fusion of the MVB membrane with the plasma membrane of the cell which results in the release of the Exo into the extracellular space [39]. It has also been shown in another study that some of the Rab family components such as Rab27A and Rab27B are the crucial mediators of Exo release. This process happens by inducing MVBs transfer to the cell periphery and finally ends with their fusion with the plasma membrane [37]. Soluble N-ethylmaleimide-sensitive factor attachment proteins receptor (SNARE) proteins are also thought to have a role in the fusion of vesicles with the plasma membrane [40]. Other molecular regulators, such as Rab11, Rab35, and cortactin, have been implicated in different steps of Exo release from different cells [38, 41–43]. Other than these regulators, Ca^2+^ levels within the cells are directly proportional to the release of Exo [44]. It has also been seen that low pH in the microenvironment affects the release of Exo and also their uptake too [45].

As mentioned above, Exo contain several signaling biomolecules and can affect target signaling pathways inside the acceptor cells. Exo can exploit several mechanisms for internalization. In short, this procedure includes the mutual interaction of exosomal ligands with cell surface receptors, membrane fusion, and endocytosis [46]. To this end, several mechanisms consisted of macropinocytosis, clathrin-, caveolin-, and lipid raft-mediated endocytosis [47]. It was suggested that the membrane distribution of specific factors such as CD9, CD81, ICAM-1, heparan sulfate proteoglycans, annexins, and integrins can affect the internalization rate of Exo [48–50]. Upon Exo uptake, these elements are internalized into early endosomes and most of the early and late endosomes are directed to fusion with lysosomes. It is thought that the degradation metabolites are then released into the cytosol and affect several signaling cascades [51].

## COPD and immune system reaction

**Fig. 2:**
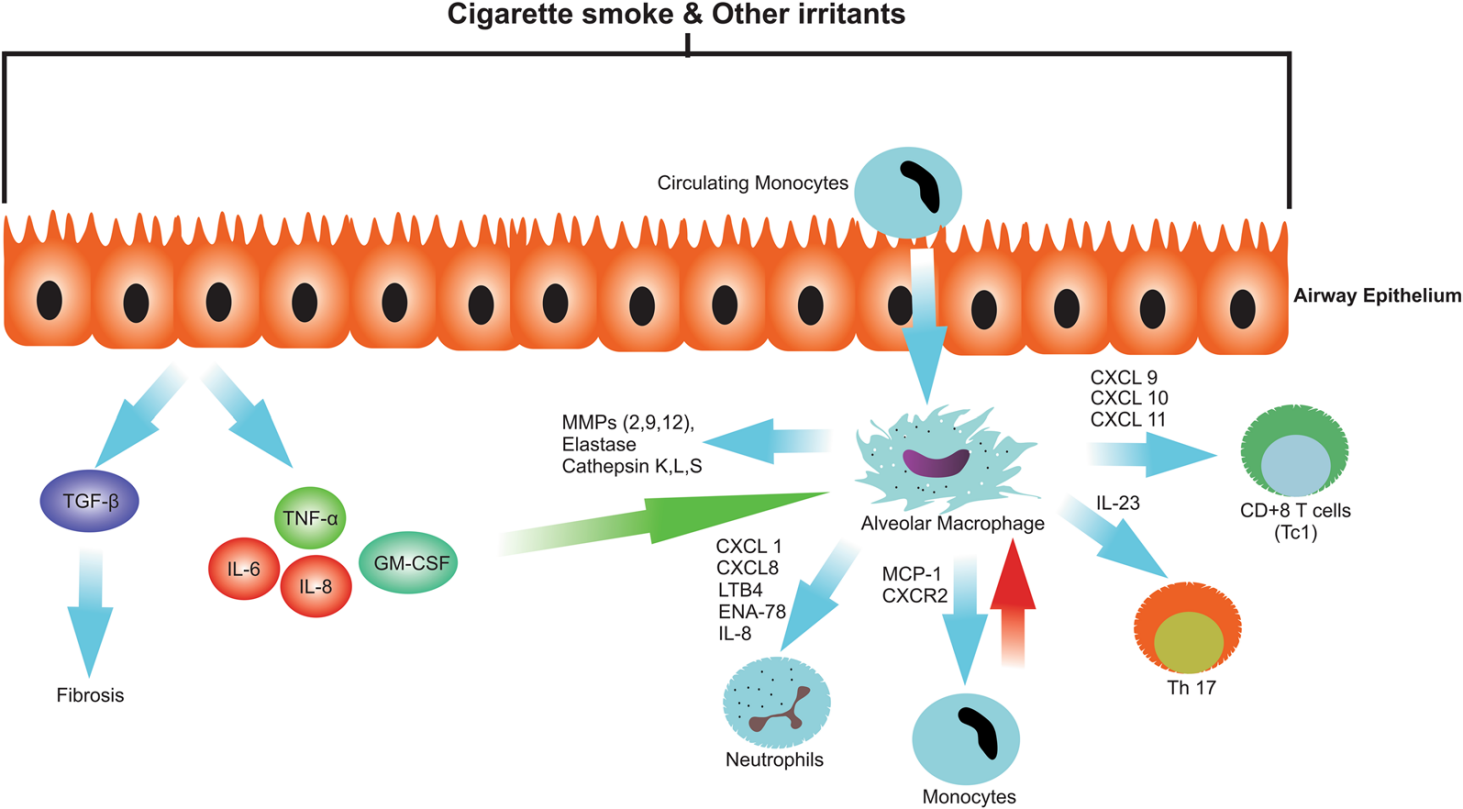
The scheme represents inflammatory mediators in COPD. Cigarette smoke and other risk factors can activate epithelial cells and also recruit macrophages from circulating monocytes to produce various chemotactic factors that attract inflammatory cells to the lung. For instance, CXCL1, CXCL8, MCP-1, LTB-4, ENA-18, and IL-8 attract neutrophils and monocytes through on CXC-chemokine receptor (CXCR) 2, monocytes also can differentiate to alveolar macrophages in the lung (red arrow). CXCL 9, 10, and 11 can attract CD+8 T cells. IL-23 derived from alveolar macrophages can also trigger th17 entrance to the lung. Recruited macrophages also secrete MMPs (2, 9, 12), elastase, cathepsin K, L, S which are involving in lung fibrosis and emphysema (More detail in Fig. 3). On the other hand, activated lung epithelial cells can secrete TGF-β which leads to fibrosis, and also TNF-α, IL-6, IL-8, and GM-CSF (GM-CSF can increase proliferation of alveolar macrophages (green arrow)). CXCL: CXC-chemokine ligand, IL: interleukin, MCP-1: monocyte chemoattractant protein 1, LTB-4: leukotriene B4, ENA-78: epithelial neutrophil activating peptide, TGF-β: transforming growth factor-beta, GM-CSF: granulocyte–macrophage colony-stimulating factor

From a clinical perspective, COPD is commonly diagnosed with progressive dyspnea, cough, and sputum production [52]. COPD is responsible for the third leading cause of human mortality globally mainly in low-income and middle-income countries [53]. It is estimated that the number of COPD deaths to increase with the aging human population shortly, while the exposure of individuals to risk factors and allergens can speed up casualties [53]. The occurrence of chronic inflammatory response and relatively irreversible changes in airway conduits, known as bronchopneumonia, bronchitis/bronchiolitis, coincided emphysema limits airflow [54]. As a correlate, treatments have been mainly focused on the modulation of immune responses [55]. Importantly, airway conduits with an internal diameter less than 2 mm are touted to be major sites for obstruction following progressive COPD [56]. The increase in airway wall thickness due to epithelial metaplasia, bronchial mucocele, hypertrophy of surrounding smooth muscle cells, and the recruitment of immune cells are common pathological findings during COPD [5]. The activation of both innate and adaptive immunity has been documented following COPD and progressive inflammation [57]. Evidence points to the local infiltration of CD8^+^ lymphocytes in air ducts [58]. There is a close association between pulmonary CD8^+^ lymphocytes and the severity of COPD [58]. As mentioned above, exposure to irritants, such as smoking, and air pollutants can intensify pathological remodeling and COPD symptoms [59, 60]. Following the exposure to irritants and allergens, the coordination of the oxidant/antioxidant system is interrupted, leading to oxidative and nitrosative stress, leading to activation of certain factors such as NF-κB and AP-1 [61–63]. The apparent accumulation of free radicals predisposes the host cells to injury via triggering apoptotic changes [64]. Several lines of documents have shown both endothelial cells (ECs) and alveolar epithelial cells displayed apoptotic changes, indicated with enhanced P53 and Caspase activity, after the onset of COPD [65, 66]. Histological examinations have indicated that pulmonary ECM like basement membranes and the interstitial matrix is degraded, leading to the lack of suitable elasticity and mechanical stability [67]. The progressive turnover in the ECM components supports prominent structural modifications and pathological remodeling [68]. Along with these changes, induction of proteolytic activity and insufficient α_1_-antitrypsin level can result in the demolition of lung parenchyma and emphysematous appearance [69, 70]. It is confirmed that infiltration of immune cells or activation of local inflammatory cells such as dust cells (alveolar macrophages) and neutrophils is associated with notable proteolytic enzymes like MMP-12, MMP-2, MMP-9, elastase, cathepsin L, and neutrophil-derived protease 3 involved in pathological remodeling (Fig. 2) [71]. In normal lungs, neutrophils are dominant inflammatory cells while the onset of chronic inflammation indicated with the elevation of pulmonary lymphocytes and macrophages that likely leads to emphysema [72, 73]. In the support of this claim, lung macrophages CD8^+^ lymphocytes are dominant inflammatory cells in the proximity of emphysematous foci [74]. Likewise, several cytokines and chemokines such as tumor necrosis factor-α (TNF-α), IL-1β, IL-6, granulocyte macrophage colony-stimulating matrix (GM-CSF), and IL-8 were actively released to the inflammatory niche [75]. The prolonged inflammatory condition results in local fibrosis by the over-production and release of tumor growth factor-β (TGF-β) from small airway epithelial cells [76]. It is important to remember that macrophages have a critical role in the development of COPD. Using ultrastructural studies, a marked increase in the number of macrophages has been indicated in pulmonary parenchyma, bronchoalveolar lavage fluid (BALF), and sputum of COPD patients [77, 78]. In addition to the proliferation of local macrophages, a large number of pulmonary macrophages are associated with enhanced monocyte recruitment to the inflamed microenvironment [79]. The apparent increase in local macrophage number is proportional to the severity of COPD. Frustrated macrophages are potent enough to release TNF-α, leukotriene B4 (LTB-4), monocyte chemoattractant protein-1 (MCP-1), reactive oxygen species, elastase, and IL-8 [80–82]. By contrast, most subsets of MMPs are released by neutrophils. Except in airway or lung parenchyma, the number of activated neutrophils is increased in sputum, bronchoalveolar lavage fluid, and airway smooth muscles of COPD patients. It would correlate with rapid transition through these tissues [83]. The expression of adhesion molecules like E-selectin on the endothelial layer can increase the intra-pulmonary entrance of blood neutrophils in COPD patients [84]. Concurrently, the concentration gradients of factors such as IL-8, LTB-4, and chemokines (C-X-C motif chemokine ligand (CXCL1) and CXCL8), and C-X-C motif chemokine 5 (ENA-78 or CXCL5) released by macrophages, T lymphocytes, and epithelial cells are involved in neutrophils chemotaxis [85]. Continuous release of IL-8, granulocyte colony stimulating factor (G-CSF), and GM-CSF can increase neutrophil's survival rate inside inflamed niches [86].

## Pathological changes following COPD

**Fig. 3:**
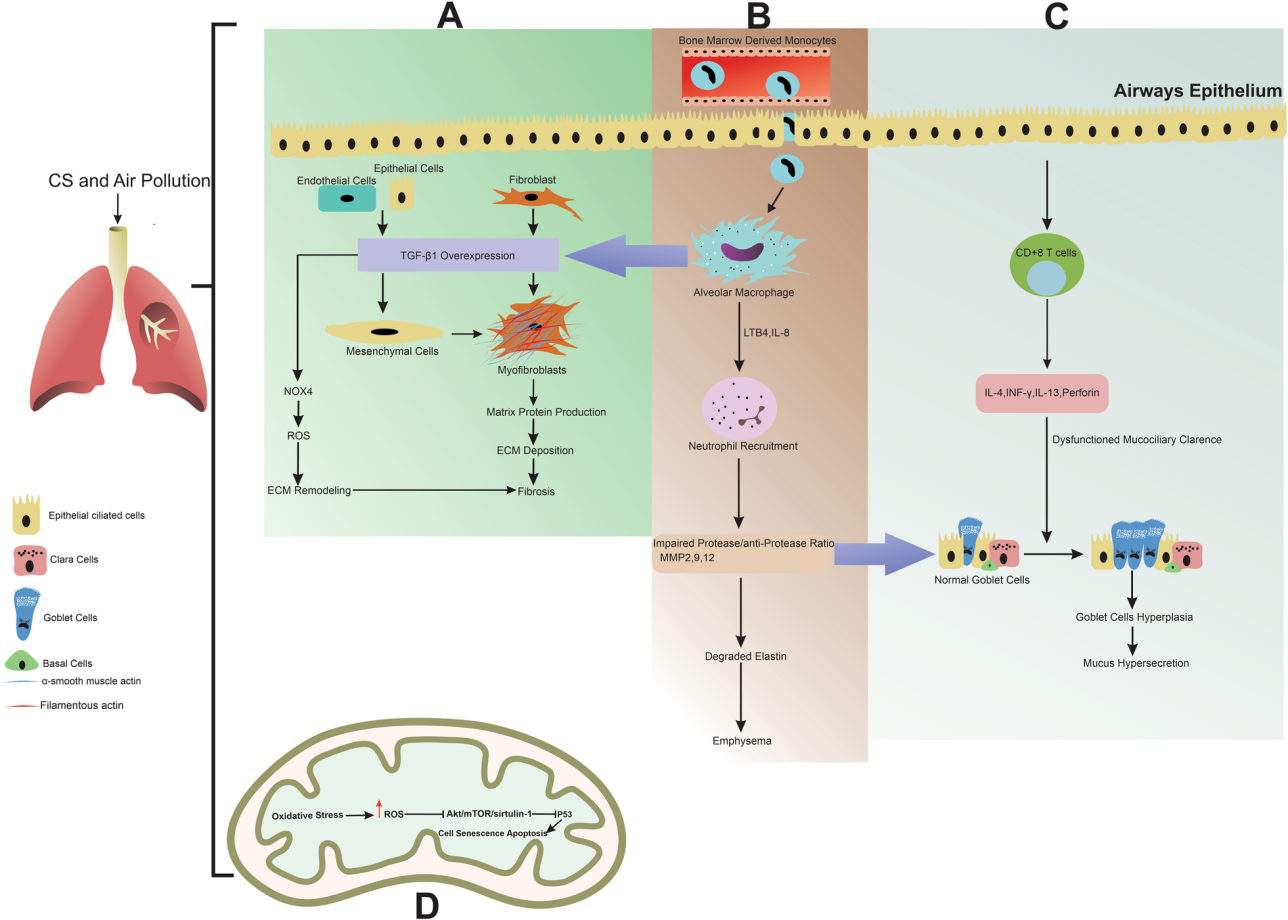
The scheme illustrates the effect of COPD on tissue remodeling. **A** Activation of alveolar macrophage leads to upregulation in TGF-β1 expression, upregulated TGF-β1 triggers differentiation of fibroblast to myofibroblasts and endothelial and epithelial cells to mesenchymal cells (EMT) which leads to fibrosis. Moreover, overexpressed TGF-β1 leads to an increase in ROS production by NOX4 activation. **B** Under inflammatory conditions, bone marrow-derived monocytes can migrate to lung tissue and differentiate to alveolar macrophages and this is, in turn, activates neutrophils in the existence of LTB-4 and IL-8. Activated neutrophils degrade elastin and as a result occurrence of emphysema through impairing protease/anti-protease balance and upregulation of MMP 2, 9, and 12; on the other hand, upregulated MMP 2, 9, and 12 induced goblet cells hyperplasia. **C** T cells derived from endothelial cells in COPD-derived inflammation-induced expression of IL-4, IFN-γ, IL-13, and perforin which leads to triggering goblet cells hyperplasia via disrupting mucociliary clearance. **D** In COPD diseases cause to increase in oxidative stress in mitochondrial which finally leads to activation of apoptosis by inhibiting P53. NOX4: NADPH oxidase 4, ROS: reactive oxygen species, ECM: extracellular matrix, LTB-4: leukotriene B4, IL: interleukin, MMPs: matrix metalloproteinase, IFN- γ: interferon-gamma

Prolonged pulmonary diseases mainly COPD are indicated with several abnormalities ranging from irreversible widespread pathological conditions in airway conduits and lung parenchyma to the alteration of broncho-pulmonary function [1]. The continuity of chronic conditions allows ciliary dysfunction, the proliferation of goblet cells, and mucous secretion [87]. Due to the airflow obstruction or narrowing, both emphysematous (excessive alveolar dilation) and atelectatic foci are detectable in gross and microscopic examinations [88]. The increased recruitment of immune cells, neutrophils, eosinophils, and further activation of alveolar macrophages coincides with the release of MMPs and lung destruction and ECM remodeling [67]. Within the lung parenchyma, the exposure of furnishing epithelial cells to pro-inflammatory cytokines leads to mitochondrial dysfunction and squamous metaplasia (Fig. 3) [89]. Recent works have established the lack of normal mitochondrial function, incomplete oxidative phosphorylation, leading to intracellular accumulation of ROS in COPD patients [90]. As a consequence, the initiation of the mitochondrial damage-associated molecular pattern (DAMPs) triggers inflammation and apoptotic changes in epithelial cells. These pathological findings are consistent with the promotion of autophagic response via mitochondrial injury which is so-called mitophagy. Ultrastructural imaging reveals the disintegration of mitochondrial membranes and localization of injured mitochondria in the periphery of the nucleus [91, 92]. These features support excessive ROS production and DNA injury. Commensurate with these descriptions, one could hypothesize that autophagic response, induced by mitophagy, can contribute to epithelial cell loss and subsequent pathologies such as apoptosis and necroptosis [93, 94]. The loss of cilia would closely relate to mitochondrial dysfunction properties as the motility of these nano-sized structures is dependent on the energy supply provided by mitochondria. The reduction of mucociliary clearance per se triggers goblet cell hyperplasia [95]. The increase of ROS was concurrent with the activation of the Akt/mTOR/sirtulin-1 axis. Sirtuin1 (SIRT-1) is a histone deacetylase linked to oxidative stress, inflammation, and cellular senescence in COPD [96, 97]. Given the highly intricate nature of COPD, the ECM network within the lung parenchyma is likely to change in COPD patients, leading to the alteration of blood-pulmonary barrier integrity. Likewise, activation of Rho-associated protein kinase and reduction of E-cadherin can lose cell-to-cell connection [98]. In the pulmonary system, ECM is the main component of basal membrane (type IV collagen and laminin) and lamina propria, and alveolar interstitium (collagens, fibronectin, elastin, and fibronectin) [99, 100]. It is thought that fibroblasts and myofibroblasts are the main ECM producers inside the pulmonary niche [101]. Reconstruction of ECM is tightly regulated by the activity of MMPs and tissue inhibitors of metalloproteinase (TIMPs) [102]. Excessive production of MMP-2, -9, and -12 increases elastin degradation and emphysema formation [103]. Histological examinations have shown less elastic fiber content in the lungs of COPD patients compared to normal tissues. [104]. In response to the reduction of elastin fibers, enhanced gene expression of elastin and fibulin-5 is normal in COPD cases [105]. The reduction of elastin is compensated with the production and deposition of type I collagen in COPD [106]. Excessive collagen leads to the loss of elasticity in alveolar structure and airway collapsibility [102].

## Role of Exo on the progression of COPD

The release of Exo by immune cells is done to maintain cell-to-cell communication while these bio-carriers can also spread the effectors associated with pathological conditions. To be specific, tissue progenitor/stem cells exit from quiescence, migrate and differentiate into the mature cell type in response to Exo released from immune cells and injured cells with pro-inflammatory response [107]. It has been shown that various types of progenitor cells can be detected within pulmonary in terms of micro-anatomical sites, specific biomarkers, and activities (Table 1). Lung progenitor cells are quiescent during the physiological condition. Shortly after the occurrence of pathological circumstances, these cells proliferate and subsequently differentiate into mature cells [108]. Among these progenitor cells, tracheal basal cells commonly express cytokeratins such as cytokeratin-14 and -5 as well as P63 [109, 110]. In response to insulting conditions such as chemical irritants (sulfur dioxide and naphthalene) and injury of the pseudostratified epithelial layer, basal cells proliferate and differentiate into both ciliated and non-ciliated luminal epithelial cells [111]. In COPD patients and cigarette smokers, an untamed proliferation of basal cells can lead to pathological hyperplasia and lack of mucociliary epithelia restoration [112]. Type 2 alveolar pneumocytes (AT2) are other lung progenitor cells with the ability to secret surfactant proteins. Under pathological conditions like inhalation of toxic gas NO_2_, proliferate and differentiate into type 1 alveolar pneumocytes (AT1) [113]. Interestingly, AT2 cells can be adversely affected by various types of respiratory disorders like idiopathic pulmonary fibrosis, leading to early cellular senescence and reduction of regenerative capacity in the pulmonary tissue [114]. Similarly, COPD can prone AT2 cells to DNA damage with the possibility of apoptotic changes [115]. Goblet cells are also known as secretory progenitor cells and produce mucus in collaboration with serous cells [116]. Like AT2 cells, goblet cells function is mainly affected by inflammation, resulting in mucus overproduction. Histological examination has shown that inflammatory compositions followed by COPD dictate goblet cells hyperplasia and consequently mucus hypersecretion [117] (Table 2).

**Table 1:** Different pulmonary progenitor cells with diverse bioactivities

Progenitor cell type	Micro-anatomical site	Specific marker (s)	Role	Ref
Basal cells	Bronchi	Cytokeratin-14, 5 and P63	Epithelium regeneration	[111]
Club/clara cells	Bronchioles	Secretory club cells-specific protein (CCSP or scgb1a1)	Involving in local inflammation response, xenobiotic metabolism	[151]
Alveolar type 2	Gas exchanging area (Alveoli)	Surfactant protein (SPC-A,B,C,D)	Surfactant secretion, tissue regeneration, differentiation to AT1	[152]

**Table 2:** Using stem cells exosomes in a variety of studies

Disease	Source of exosomes	Experiment	Results	References
Ischemia	BM-MSCs	Exo lamp2 protein fused with rabies virus glycoprotein for delivering miR-124 to promote neurogenesis following ischemia	Successful delivering of miR-124 to infarct site Inducing neuronal features in cortical neural progenitors Promoting neurogenesis	[153]
Huntington’s disease	HEK 293 cell line	Delivering miR-124 by Exo for studying on Huntington’s disease treatment	The decreasing expression of RE1-silencing transcription factor	[154]
Gliomas	BM-MSCs	Using Exo for delivering miR-124a to the treatment of gliomas	Decreasing viability and clonogenicity of glioma stem cells Increasing animal’s lifespan	[155]
Cancer	BM-MSCs	Delivering LNA (locked nucleic acid)-modified anti-miR-142-3p oligonucleotides to inhibit miR-142-3p and miR-150 expression in breast cancer cell line and mice carrying tumor	Increasing apoptosis and cytotoxicity in vitro Penetrating tumor’s barrier in vivo Decrease in tumor size and growth in vivo	[156]
Cancer	Human umbilical cord MSCs	Delivering miR-139-5p by Exo to bladder cancer cells for controlling tumorigenesis	Suppressing PRC1 expression in cancer cells and decreasing cell proliferation, migration, and invasion Decreasing the tumorigenic activity of bladder cancer cells in vitro	[157]
Cancer	HEK293T cell line	Delivering miR-204-5p by Exo and analyzing the tumor growth and chemoresistance	Increasing apoptosis Decreasing cancer cells resistance against chemotherapy drug Decreasing tumor growth	[158]
Cancer	Amniotic fluid stem cells (AFSCs)	Investigating the underlying mechanism for the effect of AFSCs on chemotherapy (CTx)-induced premature ovarian failure (POF) in woman	Exo shows anti-apoptotic features AFSCs derived Exo containing miR-10 showed preservative attitude in ovarian follicles after CTx treatment in mice	[159]
Myocardial infarction	BM-MSCs	Administrating BMSCs and BMSCs-derived Exo on myocardial infarction animal models for analyzing the function of the heart	Increasing cardiac function after injury Increasing neovascularization and myocyte survival post-injury Decreasing inflammation Decreasing apoptosis both in vitro and in vivo	[160]
Myocardial infarction	BM-MSCs	Investigating role of Exo containing miR-25-3p in cardioprotective effects against myocardial infraction	Decrease in apoptosis rate by suppression of pro-apoptotic genes Activation of suppressed cardioprotective gene eNOS and anti-inflammatory genes	
Spinal cord Injury	BM-MSCs	Using intranasally Exo loaded with phosphatase and tensin homolog siRNA (ExoPTEN) to entirely alleviate spinal cord injury	Successful migration of Exo from BBB and migrate to the spinal cord Decreasing PTEN expression in the injured spinal cord area Increasing axonal growth and neovascularization Decreasing microgliosis and astrogliosis	[161]
Osteonecrosis of the femoral head	BM-MSCs	Using siRNAs-encapsulated for analyzing its repairing effect	Increasing angiogenesis and repairing	[162]
Acute lung injury	IPSCs	Using Exo loaded with Exo to inhibit intracellular adhesion molecule-1 (ICAM-1) expression and neutrophils-endothelium (PMN-EC) adhesion	Inhibiting ICAM-1 protein synthesis Inhibiting expression of ICAM-1 surface Inhibiting PMN-EC adhesion	[163]

Whether or how inflammatory Exo or immune cell Exo can predetermine pathological remodeling and/or regeneration status need further investigations. It is also possible the molecular identity of stem/progenitor cell Exo can be different rather than that of immune cell Exo. For instance, it has been indicated that the administration of epithelial progenitor cells (EPCs) Exo in acute lung injury using lipopolysaccharide ameliorates the pathological condition. This work revealed that the regenerative effects can be associated with exosomal miRNA-126 and inhibition of sprout-related EVH1 domain-containing protein-1 (SPRED-1) via the RAF/ERK signaling axis [118]. Within the lung parenchyma, alveolar epithelial type II cells are the source of surfactant with an inherent capacity to mature into type I alveolar epithelial cells [119]. Besides differentiation into functional cells, type II epithelial cells secret a significant amount of Exo that can lead to the delivery of chemotactic factors recall mesenchymal stem cells (MSCs) under inflammatory conditions. Upon migration of MSCs into lung parenchyma, the intensity of inflammation is diminished via mitochondrial donation and improving bioenergetics [120]. This indicates that the tissue stem/progenitor cell Exo mighty blunt the pro-inflammatory condition and regenerate the injured area. However, the physiological significance of this claim is the subject of debate.

It also suggests that various immune cells like neutrophils, macrophages, resident dendritic cells (DCs), and B lymphocytes can release Exo during COPD. The activity of several enzymes, hydrolases, lysozymes, proteinases, collagenase, etc., inside neutrophils, eosinophils, and basophils granules is closely related to the tissue damage and ECM remodeling [121, 122]. In line with the active secretion of granules, the critical role of polymorphonuclear cells (PMNs) Exo has been indicated in the pathogenesis of COPD. These Exo distribute neutrophil elastase, and α_1_-antitrypsin, into the COPD inflammatory sites where active ECM destruction occurs. In vitro investigations revealed that this enzyme digests type I collagen and elastin. Noteworthy, intratracheally injection of neutrophil Exo into the mouse airway conduits led to alveolar enlargement following the ECM remodeling [123]. The close interaction of Exo-containing neutrophil elastase with type I collagen is associated with surface Mac-1 protein (integrin α_M_β_2_) [8]. In late COPD, extensive ECM destruction and pathological remodeling can be detectable. Under these conditions, the activity of specific cell lineages such as alveolar macrophages is increased as well [124]. The secretion of cytokines by alveolar macrophages into the pulmonary niche induces bronchial epithelial cells proliferation and migration. Exo isolated from alveolar macrophages are enriched in miRNA-380 a specific genetic element that tends to regulate the target cell cycle [125]. On basis of immunomodulatory properties of antigen-presenting cell Exo, such as B lymphocytes and DCs, it is postulated that these cells in line with the innate immune cell system participate in the COPD pathogenesis [126]. These immunomodulatory properties highly correlate with Exo cargo and surface antigenic molecules. For example, it has been indicated that the exosomal levels of major histocompatibility complex I and II (MHC-I and II) and heat shock proteins (70 and 90) [127]. In support of this notion, Rapaso and colleagues showed that B lymphocyte- and DC-derived Exo can activate CD4^+^ and CD8^+^ lymphocytes [128, 129]. Indeed, these Exo can harbor processed antigens to the T lymphocytes using integrins and ICAM [130]. Of note, the Exo releasing capacity of these cells can be altered according to developmental steps. For example, it has been indicated that mature DCs possess less cytosolic MVB compared to mature DCs, showing the reduction of released Exo in mature DCs. Therefore, it is logical to postulate that Exo can promote specific cell bioactivity in the target cells depending on cargo type and intracellular origination [131, 132]. Regarding the activation of T lymphocytes after exposure to DC Exo, one reason would be that DC-derived Exo contain CD86, T cell stimulator, α_M_β_2_, milk fat globule-epidermal growth factor 8, ICAM-1/CD45, and Ig family member protein [127]. Besides, molecular investigations have shown that HSP70 family member heat shock cognate protein (HSC73) is a chaperon protein involved in MHC II presentation which is abundant in DC-derived Exo [133]. Along with this protein, the content of HSP90 is also high in DC Exo, leading to immunogenicity of Exo and activation of T lymphocytes [130]. Further analyses showed that B lymphocyte Exo harbor a large amount of ICAM-1/CD45 too, late endosomal lyso-bis-phosphatidic acid, and sphingomyelin [127]. Importantly, it is noteworthy to mention that Exo from other cell types rather than immune cells can regulate pathological response in COPD patients. In the line with this claim, Xu et al. showed that Exo derived from human bronchial epithelial cells (BECs) can induce myofibroblasts differentiation through transporting overexpressed miRNA-21 in cigarette smoke-induced COPD [134]. It is thought that differentiation of bronchial fibroblasts to myofibroblasts and aggregation of myofibroblasts is one of the main reasons for the narrowing of the small airways in conditions that coincided with the inflammatory response. The abundance of specific genetic elements such as miRNA-21 in BECs Exo can lead to the deterioration of connection between BECs and fibroblasts, promoting myofibroblasts differentiation and fibrosis via modifying Von Hippel Lindau/ Hypoxia-Inducible Factor-1 (VHL/HIF-1α) signaling [134]. The increase of miRNA-21 can exacerbate COPD-related pathologies via enhanced polarization of macrophages toward M2 type and epithelial cell metaplasia via epithelial to mesenchymal transition (EMT) [135]. It seems that BECs can release Exo with distinct cargo in response to an inflammatory condition, exacerbating the conditions. The secretion of miRNA-210 via Exo can suppress autophagic response in pulmonary fibroblasts via targeting autophagy-related protein-7 (ATG7) [136]. As a correlate, prominent fibrosis and ECM remodeling occur, showing the critical role of autophagy in the regulation of COPD-related inflammatory response [137].

Taken together, the promotion of COPD can affect the production rate and exosomal cargo mainly via the alteration of the miRNA profile. Of course, the duration of disease and severity of immune responses can directly or indirectly affect exosomal content. In line with this claim, it was determined that the content of miR-122-5p was significantly reduced in bronchoalveolar fluid taken from COPD patients compared to the control group [138]. Due to the existence of active inflammatory response and recruitment of different immune cells into a pulmonary niche, it is logical to mention that the content of inflammatory Exo are high in inflamed site compared to the systemic circulation. It was suggested that sputum and bronchoalveolar lavage fluid EVs, more importantly, Exo, are indicative of inflammatory composition during the occurrence of pulmonary pathologies [139]. Of note, transcription of specific miRNAs can be modulated following the progression of different pathological conditions within the pulmonary niche. For instance, it has been shown that miR-145 and miR-338 contents were altered in conditions such as COPD, asthma, and asthma-COPD overlap syndrome, indicating that these miRNAs are common biomarkers for the detection of inflamed pulmonary tract [140]. As mentioned above, it seems that the pulmonary content of these genetic elements was higher than that of blood [141]. Whether continued inflammatory conditions can lead to balances in blood and respiratory content of specific miRNAs needs further investigation. Additionally, the specificity and sensitivity of each biomarker in response to pulmonary disease should be indicated.

## Immunomodulatory effects of stem cell Exo on COPD niche

Putative therapeutic effects of stem cells and their Exo have been proved under inflammatory conditions via juxtacrine and paracrine activities [6]. It was suggested that a part of these restorative impacts correlates with anti-inflammatory, and immunomodulatory properties of release Exo [142]. Stem cell-derived Exo are comprehensively applicable in studies ranging from pharmacological to clinical settings (Table 3). In addition to their therapeutic effects, the application of stem cell Exo as a delivery vehicle is one of the most prominent approaches in this area [143]. For instance, Fonseca and coworkers tried to deliver micro-bubbles to the respiratory tract via Exo. To this end, ultrasound signals were used to penetrate tissue to provide a way for Exo-containing micro-bubbles to reach the alveoli. They postulated that the combination of ultrasound signals and Exo administration can help in an emergency, such as COVID-19 patients, to decrease pulmonary injury [144]. It is accepted that Exo can be touted as a natural carrier for the transfer of mRNA and other genetic elements under pathological conditions. Exo can be used for the delivery of mRNA-based COVID-19 vaccines and are superior in comparison with lipid nanoparticles (LNPs) based delivery [145].

**Table 3:** Studies about the role of stem cells derived Exo on fibrosis

Disease	Source of Exosomes	Experiment	Results	References
Pulmonary fibrosis	Human umbilical cord MSCs	Using 3D cultured umbilical cord MSCs-derived Exo to treat silicosis induced lung fibrosis	Decreasing collagen I (COL1A1) and fibronectin (FN) expression -Increasing FEV0.1 amount	[164]
Renal fibrosis	BM-MSCs	Transferring miR-let7c via BM-MSCs-derived Exo to alleviate renal fibrosis	Decreasing in collagen IVα1, TGF-β1, and α-SMA expression	[165]
Liver fibrosis	BM-MSCs	Investigating the underlying mechanism for treating potential of BM-MSCs-derived Exo on liver fibrosis	Decreasing collagen aggregation and inflammation Improve the function of the liver Increase hepatocyte regeneration Responsible mechanism for the healing effect of Exo is the Wnt/β-catenin pathway	[166]
Renal fibrosis	Human umbilical cord MSCs	Investigating about repairing role of Exo though governing Yes-associated protein (YAP)	Decreasing renal fibrosis via regulating CK1δ/β-TRCP inhibited YAP activity	[167]
Cystic Fibrosis	Lung MSCs	Using lung MSCs-derived Exo to treating inflammation in cystic fibrosis	Decreasing in IL-1β, IL-8, IL-6 expression Increasing the mRNA expression of PPARγ controlling NF-kB mechanism Reducing NF-kB nuclear translocation	[168]
Cystic fibrosis	BM-MSCs	Using BM-MSCs-derived Exo containing zinc finger protein to cystic fibrosis transmembrane conductance regulator (CFTR) performance	Increasing in CFTR transcription	[169]
Hypertrophic scar (HS) fibrosis	Adipose-derived MSCs (AD-MSCs)	Studying about the effect of AD-MSCs-derived Exo in HS and its related mechanism	Suppressing proliferation and migration of HS-derived fibroblasts A decreasing expression of col 1, col 3, and α-SMA expression Increasing wound healing ratio	[170]

Up to now, there are few studies related to the application of stem cell Exo in COPD patients. In a study conducted by Maremanda et al., authors applied intraperitoneally the combination of MSCs and Exo in COPD mice. Data showed that the number of recruited neutrophils, CD4^+^ lymphocytes, and macrophages was reduced in bronchoalveolar lavage, indicating the protective role of MSCs and Exo against COPD inflammation. One reason related to the therapeutic effects of MSCs would be due to the reduction of adiponectin, keratinocytes-derived chemokine following MSCs, and Exo administration [146]. In another experiment, intra-tracheal administration of umbilical MSCs Exo in COPD rats suppressed inflammatory cytokines such as NF-κB and inhibition of alveolar septum thickening. These features coincided with the reduction of goblet cell hyperplasia compared to control asthmatic rats [147]. Like these studies, the application of placental MSCs Exo in COPD mice reduced the number of infiltrating leukocytes, diminished vascular inflammation, and suppressed local secretion of TNF-α, IL-1β, IL-12, and interferon-γ. Histological examination revealed the number of local CD80/F4^+^ macrophages in the pulmonary tract [148]. Small airway fibrosis is another complication of COPD; the underlying mechanism for this phenomenon is related to activation of myofibroblasts by the TGF-β signaling pathway [149]. One possible way to cope with this side effect might be using stem cells derived Exo; according to previous studies, effective role of this kind of Exo in fibrosis has been strongly confirmed (Table 3).

## Clinical application

Due to the pleiotropic effects of Exo from different cell sources and the complexity of underlying mechanisms in therapeutic properties of these nano-sized vesicles, there are few reports related to the application of Exo in COPD patients (Table 4). As shown in Table 3, Exo were used only for monitoring for the prediction of pathological changes and detection of valid biomarkers in COPD patients. Lack of enough knowledge related to whole Exo dose (single or repeat injection), route and time of administration, lack of standard GMP protocol, and the possibility of side effects limit the extensive application of Exo in COPD patients. Besides, standard methods have not been introduced for Exo isolation and purification, leading to heterogeneity in Exo population and content [150].

**Table 4:** Some list of clinical trials in terms of COPD and asthma recorded up to January 2022 ( available at https://clinicaltrials.gov/us)

Status	Study	Condition	Phase
Recruiting	Diagnosis of COPD via monitoring participants, including blood, urine, stool, saliva, bronchoalveolar lavage fluid	COPD	Not applicable
Recruiting	Monitoring of epigenetic, mRNA, microRNA, proteome, metabolome and microbiome alteration via exosomes, and bronchoalveolar exudates	COPD in never smoker	Not applicable
Recruiting	Monitoring exosomal non-coding RNA (ncRNA)—microRNA (miRNA), piwi-interacting RNA (piRNA) and long non-coding RNA (IncRNA) profiles	Allergic asthma and severe eosinophilic asthma	Not applicable

## Conclusion

The majority of previously published data have indicated the critical role of immune cell-derived Exo in the progression of COPD. It seems that the exosomal cargo and activity of host cells can predetermine the inflammatory/therapeutic role of Exo in specific tissue niches. In contrary to immune cell-derived Exo, stem cell-derived Exo exhibit prominent anti-inflammatory and regenerative properties under pathological conditions. Regarding the existence of few studies monitoring stem cell Exo, many comprehensive studies are needed for confirming therapeutic effects in COPD patients.

## Data Availability

Not applicable.

## Abbreviations

AP-1: Activator protein 1
ARF: Adenosine diphosphate ribosylation factor
Alix: ALG-2 interacting protein X
AT1: Alveolar type 1
AT2: Alveolar type 2
ATG7: Autophagy-related protein 7
BALF: Bronchoalveolar lavage fluid
COPD: Chronic obstructive pulmonary disease
CXCL1 CXCL8: C-X-C motif chemokine ligand
ENA-78 or CXCL5: C-X-C motif chemokine 5
DAMPs: Damage-associated molecular patterns
DCs: Dendritic cells
ESCRT: Endosomal sorting complex transport
ECs: Endothelial cells
EPC: Endothelial progenitor cell
EMT: Epithelial to mesenchymal transition
Exo: Exosome (s)
ECM: Extracellular matrix
EVs: Extracellular vesicles
G-CSF: Granulocyte colony stimulating factor
GM-CSF: Granulocyte–macrophage colony-stimulating factor
HSP: Heat shock protein
Hrs: Hepatocyte growth factor-regulated tyrosine kinase substrate
BECs: Human bronchial epithelial cells
IPF: Idiopathic pulmonary fibrosis
ICAM: Intercellular adhesion molecule
IFN-γ: Interferon-gamma
IL: Interleukin
ILVs: Intraluminal vesicles
LTB-4: Leukotriene B4
LNPs: Lipid nanoparticles
MHC I, II: Major histocompatibility complex I and II
MMP: Matrix metallopeptidase
MSCs: Mesenchymal stem cells
miRNA: MicroRNA
MVB: Microvesicular bodies
mtDAMPs: Mitochondrial damage-associated molecular patterns
MCP-1: Monocyte chemoattractant protein-1
NE: Neutrophil elastase
NF-κB: Nuclear factor-κB
PMN: Polymorphonuclear cell
ROS: Reactive oxygen species
SNARE: Soluble N-ethylmaleimide-sensitive factor attachment proteins receptor
SPRED-1: Sprout-related EVH1 domain-containing protein-1
TIMPs: Tissue inhibitors of metalloproteinase
TLR-9: Toll-like receptor-9
TGF-β: Transforming growth factor beta
TNFR1: Tumor necrosis factor receptor 1
TNF-α: Tumor necrosis factor-α
TSG101: Tumor susceptibility gene 101
VPS4: Vascular protein sorting associated protein-4
VTA1: Vesicle trafficking 1 protein
VHL/HIF-1α: Von Hippel Lindau/ Hypoxia-Inducible Factor-1
